# Trazodone for the treatment of fibromyalgia: an open-label, 12-week study

**DOI:** 10.1186/1471-2474-11-204

**Published:** 2010-09-10

**Authors:** Piedad Morillas-Arques, Carmen Ma Rodriguez-Lopez, Rocio Molina-Barea, Fernando Rico-Villademoros, Elena P Calandre

**Affiliations:** 1 Instituto de Neurociencias y Centro de Investigaciones Biomédicas, Universidad de Granada, Granada, Spain

## Abstract

**Background:**

Despite its frequent use as a hypnotic, trazodone has not been systematically assessed in fibromyalgia patients. In the present study have we evaluated the potential effectiveness and tolerability of trazodone in the treatment of fibromyalgia.

**Methods:**

A flexible dose of trazodone (50-300 mg/day), was administered to 66 fibromyalgia patients for 12 weeks. The primary outcome measure was the Pittsburgh Sleep Quality Index (PSQI). Secondary outcome measures included the Fibromyalgia Impact Questionnaire (FIQ), the Beck Depression Inventory (BDI), the Hospital Anxiety and Depression Scale (HADS), the Brief Pain Inventory (BPI), the Short-Form Health Survey (SF-36), and the Patients' Global Improvement Scale (PGI). Trazodone's emergent adverse reactions were recorded. Data were analyzed with repeated measures one-way ANOVA and paired Student's t test.

**Results:**

Trazodone markedly improved sleep quality, with large effect sizes in total PSQI score as well on sleep quality, sleep duration and sleep efficiency. Significant improvement, although with moderate effect sizes, were also observed in total FIQ scores, anxiety and depression scores (both HADS and BDI), and pain interference with daily activities. Unexpectedly, the most frequent and severe side effect associated with trazodone in our sample was tachycardia, which was reported by 14 (21.2%) patients.

**Conclusions:**

In doses higher than those usually prescribed as hypnotic, the utility of trazodone in fibromyalgia management surpasses its hypnotic activity. However, the emergence of tachycardia should be closely monitored.

**Trial registration:**

This trial has been registered with ClinicalTrials.gov number NCT-00791739.

## Background

Disturbed sleep is a prominent feature of fibromyalgia symptomatology and has been proposed as one of the core symptoms that should be systematically assessed in clinical trials for the treatment of fibromyalgia [1]. Trazodone is an old second-generation antidepressant with strong sedative activity, widely used as a hypnotic drug in sub-therapeutic antidepressant doses of 100 mg or less, although the evidence of the efficacy of this drug in treating insomnia in non-depressed patients is very limited [2]. Despite this relative paucity of data, low-dose trazodone is frequently used in fibromyalgia management to improve sleep quality [3] and is currently recommended for this purpose by the American Pain Society [4].

However, the efficacy of trazodone for fibromyalgia, either as a hypnotic or as an antidepressant, has not been adequately investigated. Only one, study published in abstract form, has evaluated the polysomnographic and clinical effects of trazodone in thirteen women with fibromyalgia [5]. The authors of the study stated that a two-month treatment with trazodone increased slow-wave sleep and reduced alpha activity but did not improve pain or psychological distress; however, they did not mention the daily dosage of trazodone that was used. The increase in slow-wave sleep induced by trazodone, in comparison with placebo treatment, has also been demostrated in two randomized clinical trials in healthy subjects [6,7]

Symptoms of depression and anxiety are frequently found in fibromyalgia patients and trazodone has antidepressant and anxiolytic properties [8] in doses higher than those usually employed to treat insomnia (i.e. 50-100 mg at bedtime) [9]. Thus, it could be useful to treat other symptoms of fibromyalgia in addition to insomnia. The present study was undertaken with the following objectives:

1. To evaluate the effectiveness of flexibly dosed trazodone in patients with fibromyalgia in improving:

- sleep quality as measured by the Pittsburgh Sleep Quality Index

- fibromyalgia overall symptomatology as measured by the FIQ total score

- anxiety, depression, and quality of life.

2. To assess trazodone's tolerability in the range of administered doses.

## Methods

The study included patients who had been diagnosed with fibromyalgia according to the American College of Rheumatology Criteria [10], were willing to discontinue their current prescribed treatment, and were referred to our unit by their physicians (general practitioners, rheumatologists and physicians from pain-management clinics). Patients who had been previously treated with trazodone but had not improved or did not tolerate the drug were excluded. Every patient gave informed consent to participation in the study, which was approved by the Ethics Committee of the University of Granada. The trial registration number was NCT-00791739.

Before starting the study the patients were required to withdraw from any current chronic pharmacological medication prescribed for fibromyalgia; no discontinuation was required for non-pharmacological treatments, herbal remedies, or drugs used on a p.r.n. basis.

After a patient-tailored washout period, trazodone was administered at a starting dose of 25 mg at bedtime and increased in 25 to 50 mg/day increments at 2-week intervals if the patient reported little or no clinical improvement. The dosage increase was maintained until a relevant clinical benefit was reached, according to the investigator's judgment, or side effects appeared. According to the trazodone package insert [11], the maximum allowed dosage was fixed at 400 mg daily. Patients were seen at baseline and at 2, 4, 6, 8, 10 and 12 weeks. Clinical investigators, all of whom were physicians, were the responsible for drug dispensation, dosage adjustments and monitoring for adverse drug reactions.

The primary outcome measure used was the Spanish-validated Pittsburgh Sleep Quality Index (PSQI) [12]. Secondary efficacy outcome measures included the following spanish-validated scales: the Fibromyalgia Impact Questionnaire (FIQ) [13], the Beck Depression Inventory (BDI) [14], the Hospital Anxiety and Depression Scale (HADS) [15], the Brief Pain Inventory (BPI) [16], the Short-Form Health Survey (SF-36) [17], and the Patients' Global Improvement Scale (PGI). Scales were administered at baseline and at weeks 6 and 12, with the exception of the SF-36 which was administered only at baseline and at week 12. Trazodone emergent side effects were recorded by the physician at each patient's visit by means of an open-ended question; patients were also instructed to phone the attending physician if they believed that they were experiencing any drug-related side effect.

The intention-to-treat (ITT) sample included those patients who started trazodone and had at least a post-baseline assessment. Data were analyzed with repeated measures ANOVA and with paired Student's t test in the case of SF-36. Effect sizes were calculated according to Cohen's formula.

## Results

**Figure 1:**
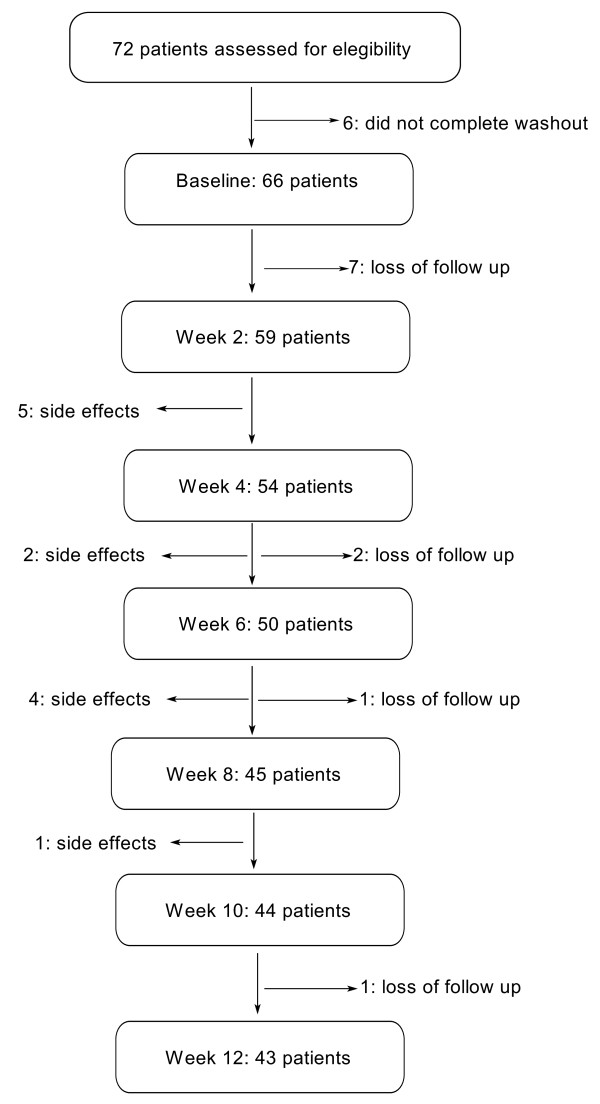
**Patients flow diagram**.

As shown in Figure 1, a total of 72 patients willing to participate were screened. However, 6 of them were unable to complete the washout period. Therefore 66 patients, 61 women and 5 men aged from 22 to 70 years (48 ± 9.7), began trazodone treatment. Fifty nine patients completed at least a one post-baseline assessment and constituted the ITT sample. Twenty three patients (35%) withdrew from the study at different times during the evaluation period, 12 of them due to treatment-emergent side effects, and 11 were lost to follow up. Among the 66 patients who started trazodone, 33 (59%) had been prescribed benzodiazepines, zolpidem or zopiclone on a daily basis but still complained of sleep difficulties. Final doses of trazodone varied from 50 to 300 mg/day, with a mean value of 200 ± 70 mg/day.

Efficacy measure data are shown in Tables 1, 2 and 3. Trazodone had a significant effect on sleep parameters with the largest effect sizes in sleep quality, duration and efficacy at week 12 (Table 1). Improvement in sleep quality, as measured by the differences in PSQI total scores between week 12 and baseline was not related with the final dose of trazodone.

**Table 1:** PSQI total and individual scores throughout the study period

	baseline	week 6	week 12	P (anova)
Total score (mean ± s.d.)	16.2 ± 3.3	12.9 ± 4.1***	12.1 ± 5.0***	< 0.0001
ES		**1.00**	**1.24**	
Sleep quality (mean ± s.d.)	3.58 ± 0.5	1.78 ± 0.9***	1.64 ± 0.9***	< 0.0001
ES		**3.6**	**3.88**	
Sleep latency (mean ± s.d.)	2.49 ± 0.8	2.2 ± 0.9**	1.9 ± 1.1***	< 0.0001
ES		0.36	**0.73**	
Sleep duration (mean ± s.d.)	2.58 ± 0.6	1.98 ± 1.0***	1.75 ± 1.1***	< 0.0001
ES		**1.00**	**1.38**	
Sleep efficiency (mean ± s.d.)	2.68 ± 0.7	2.03 ± 1.2***	1.59 ± 2.2***	< 0.0001
ES		**0.93**	**1.56**	
Sleep disturbance (mean ± s.d.)	2.36 ± 0.5	2.07 ± 0.6**	2.02 ± 0.7***	= 0.0001
ES		**0.58**	**0.68**	
Sleeping medications (mean ± s.d.)	1.42 ± 1.5	0.97 ± 1.4*	0.73 ± 1.2**	= 0.0033
ES		0.30	0.46	
Daytime dysfunction (mean ± s.d.)	2.32 ± 0.8	2.02 ± 0.9**	1.98 ± 0.9**	= 0.0013
ES		0.38	0.42	

*: p < 0.05, **:p < 0.01, and ***: p < 0.001 in relation to baseline (Dunnett's post-test)

ES: effect size; bold: moderate and large effect sizes

**Table 2:** Fibromyalgia Impact Questionnaire scores throughout the study period

	baseline	week 6	week 12	P (anova)	
Total score (mean ± s.d.)	77.5 ± 13.4	70.8 ± 15.8***	67.9 ± 17.9***	< 0.0001	
ES		**0.5**	**0.72**		
Work (mean ± s.d.)	8.15 ± 1.6	7.42 ± 2.0**	7.43 ± 2.1**	= 0.0014	
ES		0.46	0.45		
Pain (mean ± s.d.)	8.26 ± 1.7	7.78 ± 1.7*	7.46 ± 1.9***	= 0.0007	
ES		0.28	0.47		
Fatigue (mean ± s.d.)	8.75 ± 1.4	8.09 ± 2.1*	8.03 ± 2.1*	= 0.0140	
ES		0.47	**0.51**		
Morning tiredness (mean ± s.d.)	8.86 ± 1.4	7.78 ± 2.4**	7.72 ± 2.6**	= 0.0005	
ES		**0.77**	**0.81**		
Stiffness (mean ± s.d.)	8.04 ± 2.0	7.12 ± 2.8**	6.94 ± 2.8***	= 0.0006	
ES		0.46	**0.55**		
Anxiety (mean ± s.d.)	8.15 ± 1.9	7.44 ± 2.7*	7.04 ± 2.7***	= 0.0010	
ES		0.37	**0.58**		
Depression (mean ± s.d.)	7.94 ± 2.5	6.98 ± 2.9	7.06 ± 3.0	= 0.0027	
ES		0.38	0.35		

*: p < 0.05, **:p < 0.01, and ***:p < 0.001 in relation to baseline (Dunnett's post-test)

ES: effect size;bold: moderate and large effect sizes

**Table 3:** Depression, anxiety, pain and quality of life scores throughout the study period

	baseline	week 6	week 12	P (anova)
BDI-whole sample (mean ± s.d.)	28.0 ± 10.7	24.9 ± 12.3*	22.5 ± 10.7***	< 0.0001
ES		0.29	**0.51**	
BDI > 18 (N = 41) (mean ± s.d.)	31 ± 9.2	26.9 ± 12.0*	23.3 ± 10.1***	< 0.0001
ES		0.45	**0.84**	
HADS-depression (mean ± s.d.)	12.4 ± 4.9	11.6 ± 4.8	11.0 ± 5.0**	= 0.0142
ES		0.16	0.29	
HADS depression > 7 (N = 43) (mean ± s.d.)	13.6 ± 3.8	12.5 ± 4.2	11.6 ± 4.5**	= 0.0054
ES		0.29	**0.53**	
HADS anxiety (mean ± s.d.)	13.8 ± 4.2	13.1 ± 4.7	12.3 ± 4.9**	= 0.0041
ES		0.17	0.36	
HADS anxiety >7 (N = 47) (mean ± s.d.)	14.5 ± 3.4	13.6 ± 4.3	12.7 ± 4.4**	= 0.0061
ES		0.26	**0.53**	
BPI: mean pain severity (mean ± s.d.)	7.47 ± 1.6	7.00 ± 1.8*	6.95 ± 1.8*	= 0.0154
ES		0.29	0.33	
BPI: pain interference with daily activities (mean ± s.d.)	8.42 ± 1.7	7.11 ± 2.2***	6.82 ± 2.6***	< 0.0001
ES		**0.66**	**0.84**	
SF-36: PCS (mean ± s.d.)	27.3 ± 4.1		28.2 ± 5.2	N.S.
ES			-0.22	
SF-36: MCS (mean ± s.d.)	28.0 ± 11.5		30.7 ± 12.5*	= 0.0383
ES			-0.23	

*: p < 0.05, **:p < 0.01, and ***: p < 0.001 in relation to baseline (Dunnett's post-test)

ES: effect size; bold: moderate and large effect sizes

The FIQ total scores, whose baseline values ranged from 33 to 99, decreased significantly, with moderate to large effect sizes in fatigue, morning tiredness, stiffness and anxiety at week 12 (Table 2). There was not a clear-cut relationship between trazodone dose and differences between FIQ total scores at week 12 and at baseline; however, patients receiving >200 mg/day of trazodone improved significantly more than those receiving doses of 100 mg or less (mean FIQ scores differences of 11.6 ± 2.7 and 4.1 ± 1.9 respectively, p = 0.029).

Both depression and anxiety scores were significantly lower at week 12, especially among patients with clinically relevant depression and/or anxiety at baseline (Table 3). Similarly to the FIQ findings, the differences between BDI total scores at week 12 and baseline were significantly higher in patients receiving >200 mg/day of trazodone than in those receiving doses of 100 mg or less (mean scores differences of 8.3 ± 2.2 and 2.1 ± 0.9 respectively, p = 0.015).

Trazodone did not markedly improve pain intensity but had a significant effect on pain interference with daily activities at week 12 (Table 3). A moderate improvement, although with a small effect size, was also seen in the Mental Component Summary (MCS) of the SF-36 at week 12.

Among the 43 patients that completed the study, 8 (18.6%) reported to much or very much improvement under trazodone; 20 (46.5%) reported slight improvement, 8 (18.6%) reported no change, and 7 (16.3%) reported worsening.

The most frequent side effect of trazodone was tachycardia (N = 14, 21.2% of the total sample), followed by dry mouth (N = 10, 15.2%), sedation (N = 7, 10.6%), dizziness and lightheadedness (N = 6, 9.1% for each adverse reaction). Twelve (18.2%) patients withdrew from the study due to side effects, six (50%) of them due to tachycardia; all of these patients were receiving trazodone doses equal to or lower than 150 mg daily.

## Discussion

As expected, trazodone had the greatest impact on sleep parameters. In addition, although to varying degrees, significant improvements were also observed in general fibromyalgia symptomatology as well as in anxiety and depression. Branco et al [5] did not find that trazodone caused changes in psychological profile nor clinical symptoms under trazodone treatment. However, as stated in the introduction, their abstract did not specify tradozone dosage. Furthermore, the study lasted only two months which was probably too short a time period of time too short to appraise the full efficacy of the drug, since, as the STAR*D study has demonstrated [18], depressed patients may need a treatment course longer than 8 weeks to achieve remission under antidepressant treatment.

The improvement of sleep quality with trazodone was striking, especially when considering that most patients previously received benzodiazepines, zolpidem or zopiclone with only limited success. Benzodiazepines, zolpidem, zopiclone and trazodone all increase total sleep time; the main difference is that trazodone increases stages 3 and 4 of sleep whereas the other drugs mainly increase stage 2 of sleep [19,20]. Patients with fibromyalgia show a reduction in the total duration of slow wave sleep, and intrusion of alfa waves superimposed to the delta frequency which is characteristic of the slow wave sleep [21]. Trazodone does not only increase the total duration of slow wave sleep, but has also demonstrated to reduce the alpha activity in the sleep of fibromyalgia patients [5]. It is thus possible that the beneficial effects of trazodone on sleep quality in our patients could be due to its differential effects on sleep structure.

Bodily pain, which is the cardinal symptom that defines fibromyalgia, was only slightly improved with trazodone administration; however, other relevant symptoms of the disease such as fatigue, morning tiredness, and stiffness were alleviated, and there was also a strong beneficial effect on sleep parameters. This clinical profile likely does not warrant the use of trazodone use as a single agent in fibromyalgia treatment at the low-to-medium trazodone doses evaluated in our study but suggests that it could be used as an adjunctive agent. Although monotherapy would be an ideal option for the treatment of fibromyalgia, this is not possible in many cases. The complexity of fibromyalgia symptomatology has been acknowledged, and a Symptom Severity Scale for the disease has recently been included in the new proposal of fibromyalgia diagnostic criteria the by the American College of Rheumatology [22]. Patients with a wide range of symptoms frequently require the use of more than one drug simultaneously, and the need for the prescription of two or more drugs for fibromyalgia management has been acknowledged [23]. In fact, several observational prospective and longitudinal studies show that prescription drugs use is high among fibromyalgia patients [24], and that polytherapy is usual, with a mean range of 2.7 to 3.3 drugs per patient [22,25,26]. Given its positive effect on sleep quality, depression and anxiety, and its lack of analgesic activity, trazodone could be combined with drugs that have analgesic efficacy but with less favourable effects on sleep parameters, such as NSAIDs, tramadol, or duloxetine [27-30].

Decreases in PSQI total scores were not related with the final dose of trazodone, suggesting that the improvement in sleep quality associated with trazodone treatment can be reached with low daily doses of the drug. In contrast, the highest trazodone doses (i.e., those higher than 200 mg daily) were associated with more pronounced decreases both in FIQ and BDI total scores in some patients. These data seem to indicate that, in doses similar to those used for the treatment of depression, trazodone could be also useful to treat fibromyalgia symptomatology.

Among our patients, tachycardia was the most frequent adverse reaction and the main reason for treatment discontinuation. It was seen at drug doses as low as 50 mg/day and usually appeared during the first weeks of treatment. Tachycardia is a relatively uncommon side effect of trazodone [11,31], having been reported in clinical trials in percentages ranging from 0 to 7% of patients. This unexpectedly high prevalence of tachycardia in our sample might be due to the well-known association between fibromyalgia and autonomic dysfunction, which has been shown to facilitate increases in heart rate as a response to different kinds of stimuli [32].

The main limitation of our work was its uncontrolled and open-label design which may have overestimated treatment effects. However, the study was conceived as a preliminary research aiming to evaluate the potential usefulness of an old drug, widely used but scarcely investigated, in the management of fibromyalgia. The 12-week duration of the study may be an additional limitation; a longer evaluation period may have been more appropriate, particularly considering that the open-label design of the study did not allow us to control for a potential placebo effect.

The reduction of the sample size due to patients withdrawal may have reduced representativeness of sample and the generalizability of results. However, since we used an intent-to-treat approach to the analysis, it does not seem probable that this fact affected the external validity of the study in a relevant manner.

The use of an open-ended question to assess emergent side effects may have underestimate the their actual frequency; this is, however, the standard method of evaluation of adverse reactions in clinical trials, and unexpected and/or serious side-effects are usually captured by this kind of evaluation. On the other hand, the use of a checklist could have had the opposite effect, i.e. to overestimate the frequency of side effects.

Finally, the possibility that the washout period could have increased patients expectations in relation to trazodone treatment cannot be overlooked. Nevertheless, washout was essential to avoid side effects due to drug interactions and its duration was kept as short as possible, not lasting more than four weeks as maximum in the case of patients heavily polymedicated.

Despite the above-mentioned limitations of the study, our data suggest that trazodone deserves further evaluation as a potential treatment for fibromyalgia. Additional studies exploring the efficacy of the drug in doses up to 400 mg/day, with a randomized and controlled design, would be worthy to be performed in the future.

## Conclusion

Despite the limitations of its uncontrolled design, the results of our study show that trazodone markedly improves subjective sleep quality in patients with fibromyalgia. The beneficial effects of trazodone seem also to extend to other symptoms such as anxiety and depressive symptoms. However, its usefulness seems to be limited by its tolerability, tachycardia being a relevant adverse reaction that should be closely monitorized.

## Competing interests

The authors declare that they have no competing interests.

## Authors' contributions

EPC and FRV designed the study, evaluated the results, and wrote the manuscript. PMA, CMRL and RML did the clinical work. All authors have read and approved the final manuscript

## Pre-publication history

The pre-publication history for this paper can be accessed here:

http://www.biomedcentral.com/1471-2474/11/204/prepub
